# Analysis of Differentially Expressed Genes in Endothelial Cells Following Tumor Cell Adhesion, and the Role of PRKAA2 and miR-124-3p

**DOI:** 10.3389/fcell.2021.604038

**Published:** 2021-02-19

**Authors:** Yan Pan, Marhaba Abdureyim, Qing Yao, Xuejun Li

**Affiliations:** ^1^Department of Pharmacology, Health Science Center, School of Basic Medical Sciences, Peking University, Beijing, China; ^2^Department of Biochemistry and Molecular Biology, Ningxia Medical University, Yinchuan, China

**Keywords:** tumor-endothelial adhesion, PRKAA2, miR-124-3p, long non-coding RNA, bioinformatic analysis

## Abstract

Tumor cell adhesion to the endothelium is one pattern of tumor–endothelium interaction and a key step during tumor metastasis. Endothelium integrity is an important barrier to prevent tumor invasion and metastasis. Changes in endothelial cells (ECs) due to tumor cell adhesion provide important signaling mechanisms for the angiogenesis and metastasis of tumor cells. However, the changes happened in endothelial cells when tumor–endothelium interactions are still unclear. In this study, we used Affymetrix Gene Chip Human Transcriptome Array 2.0. and quantitative real-time PCR (qPCR) to clarify the detailed gene alteration in endothelial cells adhered by prostate tumor cells PC-3M. A total of 504 differentially expressed mRNAs and 444 lncRNAs were obtained through chip data analysis. Gene Ontology (GO) function analysis showed that differentially expressed genes (DEGs) mainly mediated gland development and DNA replication at the biological level; at the cell component level, they were mainly involved in the mitochondrial inner membrane; and at the molecular function level, DEGs were mainly enriched in ATPase activity and catalytic activity. Kyoto Encyclopedia of Genes and Genomes (KEGG) signal pathway analysis showed that the DEGs mainly regulated pathways in cancer, cell cycle, pyrimidine metabolism, and the mTOR signaling pathway. Then, we constructed a protein–protein interaction functional network and mRNA–lncRNA interaction network using Cytoscape v3.7.2. to identify core genes, mRNAs, and lncRNAs. The miRNAs targeted by the core mRNA PRKAA2 were predicted using databases (miRDB, RNA22, and Targetscan). The qPCR results showed that miR-124-3p, the predicted target miRNA of PRKAA2, was significantly downregulated in endothelial cells adhered by PC-3M. With a dual luciferase reporter assay, the binding of miR-124-3p with PRKAA2 3’UTR was confirmed. Additionally, by using the knockdown lentiviral vectors of miR-124-3p to downregulate the miR-124-3p expression level in endothelial cells, we found that the expression level of PRKAA2 increased accordingly. Taken together, the adhesion of tumor cells had a significant effect on mRNAs and lncRNAs in the endothelial cells, in which PRKAA2 is a notable changed molecule and miR-124-3p could regulate its expression and function in endothelial cells.

## Introduction

Tumor metastasis is the main cause of cancer-related death in humans. It is a complex process that involves a wide variety of cell–cell communication (HDaF, 1996). According to the “seed and soil” hypothesis, primary tumor cells first invade the surrounding extracellular matrix (ECM) and stromal cell layers, intravasate into the bloodstream, and then survive and disseminate in the circulation and reach distant organs (Fidler, 2003). During extravasation, the intact endothelium serves as a defensive barrier to prevent the extravasation of tumor cells. During this complex cascade of events, tumor cells have shown an ability to induce endothelial changes by directly targeting cells via soluble factors, adhesion receptors, gap junctions, and vesicles (Lopes-Bastos et al., 2016). Evidence has been provided that tumor cells can increase the number of dead endothelial cells when they were co-cultured and that tumor cell-induced endothelial necroptosis promotes tumor cell extravasation and metastasis, leaving gaps in the endothelial barrier (Strilic et al., 2016). Additionally, tumor-induced activation of quiescent endothelial cells involves the expression of angiogenesis-related receptors and the induction of autocrine growth loops (Khodarev et al., 2003). Hence, investigating the underlying molecular mechanisms and biological processes during tumor–endothelium interaction is important to understand and control tumor metastasis.

In recent years, genomics, transcriptomics, and proteomics analyses have been widely used in cancer-related research. Among them, transcriptomics includes analysis of all RNA from tissue or cells, revealing the molecular mechanisms of specific biological processes and disease by studying gene transcription and function. Long non-coding RNAs (lncRNAs) are RNA molecules greater than 200 nucleotides in length that exert their functions through interactions with other components, such as protein, RNA, and DNA (Guttman and Rinn, 2012). Recent studies showed that lncRNAs play key roles in the initiation and progression of cancer (Liu et al., 2014; Chen et al., 2019; Zhou et al., 2019) via sponging miRNAs as competing endogenous RNA (Salmena et al., 2011) (ceRNA) and consequently regulating the mRNA expression level. However, the mRNA and lncRNA expression profiles in tumor–endothelium interaction are still unknown. In order to identify the aberrantly expressed mRNAs and lncRNAs in endothelial cells adhered by prostate tumor cells PC-3M, we screened expression profiles in the tumor–endothelium adhesion model with the Human Gene 2.0 ST GeneChip^®^ array. Based on these data, we conducted an integrated bioinformatics analysis to clarify the molecule mechanism during this tumor–endothelium interaction.

## Materials and Methods

### Cell Culture

Human umbilical vein endothelial cells (HUVECs, Lifeline Cell Technology, MD, United States) were cultured in M199 (Gibco; Grand Island, NY, United States) supplemented with 20% heat-inactivated fetal bovine serum (FBS), 20 μg/ml endothelial cell growth supplement (Sigma, St. Louis, MO, United States), 100 U/ml penicillin, 100 μg/ml streptomycin, and 2 mM L-glutamine. The identity of the HUVECs was confirmed by their polygonal morphology and expression of factor VIII-related antigen. PC-3M cells were cultured in RPMI 1640 medium containing 10% heat-inactivated FBS, 100 U/ml penicillin, and 100 μg/ml streptomycin in a humidified incubator with 5% CO_2_ in air at 37°C (Yan Pan et al., 2011).

### Tumor–Endothelium Adhesion Model

HUVECs were cultured in 96-well dishes coated with 1% gelatin. When HUVECs reached an approximate confluence of 100%, PC-3M cells were washed and resuspended in culture medium (10^5^ cells/ml). One hundred microliters of the tumor cell suspension was added to HUVEC monolayers in 96-well dishes and cocultured with HUVECs for 30 min at 37°C. At the end of the incubation, the wells were washed three times with PBS to remove adherent and non-adherent PC-3M cells (Andrews et al., 2001).

### RNA Preparation and Microarray Profiling

For Affymetrix microarray profiling, total RNA was isolated with TRIzol reagent (Invitrogen, Canada) and purified using an RNeasy Mini Kit (Qiagen, Germany), including a DNase digestion treatment. RNA concentrations were determined by the absorbance at 260 nm, and the quality control standards were A260/A280 = 1.8–2.1, using NanoDrop 2000 (Thermo, America). The cDNA of PC-3M-adhered HUVECs (TC-EC) or from HUVEC controls (EC) was hybridized to Human Gene 2.0 ST GeneChip^®^ arrays (Affymetrix, America) according to the user manuals. Affymetrix^®^ Expression Console software (version 1.2.1) was used for the microarray analysis.

### Analysis of Differential mRNAs and lncRNAs

Raw data (CEL files) were normalized at the transcript level using the robust multi-average method (RMA workflow). Median summarization of transcript expressions was calculated. Gene-level data were then filtered to include only those probe sets that are in the “core” metaprobe list, which represent RefSeq genes. The primary data were uploaded to the GEO database, and the affiliation number is GSE162957. Student’s *t*-test was used to calculate the *P*-value and false discovery rate (FDR) and evaluate the expression differences of mRNAs and lncRNAs between TC-EC and EC. *P* < 0.05, FDR < 0.05, and | log_2_FC| > 1 were considered differentially expressed mRNAs or lncRNAs.

### Gene Ontology and Pathway Analysis

To understand the underlying biological processes between differentially expressed mRNAs, the gene ontology (GO) database by DAVID (Database for Annotation, Visualization, and Integrated Discovery)^1^ was used to perform functional enrichment analysis. Pathway analysis was conducted using the Kyoto Encyclopedia of Genes and Genomes (KEGG)^2^. The relationships among the enriched clusters from the GO and KEGG pathway analyses were visualized using R ggplot2 package and Cytoscape v3.7.2.

### Differential mRNA–mRNA Interaction Network

Using the KEGG database, we constructed a differentially expressed mRNA interaction network aiming at studying the molecule interaction in the TC-EC model. Cytoscape software v3.7.2. was used to construct and visualize the network. In the network, the degree of a gene was defined as the number of directly linked genes within a network, which could assess the relative significance of a gene within the network. Meanwhile, the character of a gene was also described by betweenness centrality, which was an indicator of a gene’s centrality in a network. Betweenness centrality was equal to the number of shortest paths from all of the vertices to all of the others that passed through that gene (Zhang and Wiemann, 2009). Thus, degree and betweenness centrality were used as two indicators to identify the most important genes (Feng et al., 2016).

### mRNA–lncRNA Co-expression Network

The mRNA–lncRNA expression correlation networks in the TC-EC model and in EC alone were constructed using Affymetrix microarray profiling data. To assess network characteristics, we computed the degree of each node in both networks. We compared the values of each group and subtracted them to get the | Diff| value. Both mRNA–lncRNA co-expression correlation networks were constructed using the top 10 mRNAs/lncRNAs arranged from large to small and visualized using Cytoscape v3.7.2. Those mRNA/lncRNAs without interaction relationships were not displayed in the network.

### Prediction of miRNAs Targeted at PRKAA2

The predicted miRNAs targeted at PRKAA2 mRNA were obtained from the miRDB database^3^, RNA22 database^4^, and Targetscan^5^. The binding sites of miR-124-3p within the 3’UTR of PRKAA2 mRNA were obtained using Targetscan.

### Lentivirus Infection

The pFU-GW (hU6–MCS–ubiquitin–EGFP–IRES–puromycin) vector was used for the knockdown of miR-124-3p. The mature microRNA in cells can be competitively bound by antisense microRNA sequences, thus affecting the binding between the mature microRNA and target gene mRNA and reducing the inhibition of the microRNA on the translation of target gene mRNA. The infection of antisense miR-124-3p sequences (GGCATTCACCGCGTGCCTTA) was done according to Shanghai GeneChem Corporation’s operation manual. Target cells at the logarithmic growth stage were digested by trypsin to make a cell suspension. The cell suspension (cell number was about 5 × 10^4^) was inoculated in a 6-well plate and cultured in an incubator with 5% CO_2_ at 37°C until the cell fusion rate reached about 30%. An appropriate amount of virus was added according to the MOI value of cells. After 12 h, cells were observed and it was confirmed that the virus had no obvious cytotoxic effect. The culture medium was changed after 24 h. If there were significant cytotoxic effects, the medium was replaced immediately. The expression of the reporter gene EGFP on lentivirus was observed 3 days after infection. The cells with a fluorescence rate of more than 80% were divided into two parts. One part was placed in a 12-well culture plate to be overgrown and then collected for RNA extraction; the other part was placed in a 6-well plate to be overgrown and then collected for immunofluorescence detection to determine the infection efficiency.

### Quantitative Real-Time PCR

Total RNA was extracted using the TRIzol reagent (Invitrogen, CA, United States), and it was reverse-transcribed into cDNAs using the M-MLV reverse transcriptase (Promega, Madison, United States) with Oligo (dT18) RT primers (Sangon, Shanghai, China). Quantitative real-time PCR was carried out using SYBR Master Mixture (TAKARA, Dalian, China). The relative expression levels of the mRNAs were normalized vs. GAPDH expression, and miRNAs were normalized to U6. Comparative quantification was performed using the 2^–△^^△^^C^t method. The primer sequences are listed below. (The miRNA primers were purchased from RiboBio Corporation and sequences are displayed by their article number).

### Dual Luciferase Reporter Assay

For the luciferase assays, the wild-type and mutant PRKAA2 3’UTR and the miR-124-3p were amplified by PCR. Luciferase reporter plasmids pGL3-PRKAA2-3’UTR-WT, pGL3-PRKAA2-3’UTR-MUT, and pcDNA3.1(-)-miR124-3p were prepared. The primer sequences for the amplification of PRKAA2 3’UTR were as follows: PRKAA2 3’UTR forward: GATCGCCGTGTAATTCTAGAGTGTTGTAAACTC TGAATGTG, PRKAA2 3’UTR reverse: CCGGCCGC CCCGACTCTAGATTAGAGTTCACAGTTCTACAACAAC; The 293T cells were seeded in 24-well plates at a density of 10^5^ cells/well. After 24 h culture, the plasmids were transfected into 293T cells with the indicated pGL3 firefly luciferase vector and pGL3 Renilla luciferase vector. After 48 h, cells were lysed and luciferase activity was measured using a dual-luciferase reporter assay system (Promega, United States) according to the manufacturer’s protocol. Data were normalized to the Renilla luminescence and presented relative to miRNA-124-3p-NC transfected group.

### Statistical Analysis

The data are presented as means ± *SD*. Two-tailed Student’s *t*-tests and one-way analysis of variance (ANOVA) were used to compare the differences between groups using Graphpad Prism 8.0 software. *P* < 0.05 was considered to be statistically significant.

## Results

### mRNA and lncRNA Expression Profile of TC–EC Interaction

**FIGURE 1:**
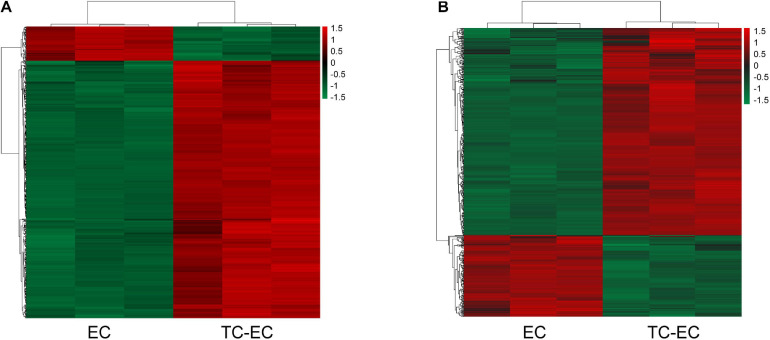
Hierarchical clustering of aberrant expressed mRNAs **(A)** and lncRNAs **(B)** detected in the TC–EC model and EC alone. Every column represents a cell sample, and every row represents an mRNA or lncRNA probe. Red color indicates overexpression genes and green color indicates low-expression genes.

A total of 504 mRNAs and 444 lncRNAs were identified to be differentially expressed (*P*-value < 0.05, | log_2_FC| > 1) between the TC–EC model and EC alone, including 445 upregulated and 59 downregulated mRNAs (Figure 1A) and 328 upregulated and 116 downregulated lncRNAs (Figure 1B). The lists of the top 20 differentially upregulated (Table 1) and downregulated mRNAs (Table 2) in the large to small arrangement of | log_2_FC| are provided. Combined with the results above, the adhesion of tumor cells significantly dysregulated the mRNAs and lncRNAs in endothelial cells.

**TABLE 1:** The list of top 20 differentially up-regulated mRNAs arranged from large to |log_2_FC| values.

**Gene symbol**	**|log_2_FC|**	***P*-value**	**FDR**	**Chromosome**
LGR5	4.665	<1e–07	<1e–07	chr12
IL13RA2	4.505	<1e–07	<1e–07	chrX
IF130	4.457	<1e–07	<1e–07	chr19
SLPI	4.161	<1e–07	<1e–07	chr20
ANKRD30A	4.146	<1e–07	<1e–07	chr10
NEFL	4.104	<1e–07	<1e–07	chr8
MMP13	4.041	<1e–07	<1e–07	chr11
DPP4	4.037	<1e–07	<1e–07	chr2
POF1B	3.936	<1e–07	<1e–07	chrX
RPS6KA6	3.696	<1e–07	<1e–07	chrX
BMPR1A	3.596	<1e–07	<1e–07	chr10
SEMA3D	3.437	<1e–07	<1e–07	chr7
GPR98	3.409	<1e–07	<1e–07	chr5
PRAC	3.359	<1e–07	<1e–07	chr17
SERPINB5	3.265	<1e–07	<1e–07	chr18
DSG2	3.263	<1e–07	<1e–07	chr18
TOMIL1	3.254	<1e–07	<1e–07	chr17
NELL2	3.168	<1e–07	<1e–07	chr12
GPR110	2.995	<1e–07	<1e–07	chr6
PRKAA	2.975	<1e–07	<1e–07	chr1

**Table:** 

**Genes**	**Forward primer**	**Reverse primer**
GAPDH	TGACTTCAACAGCGACACCCA	CACCCTGTTGCTGTAGCCAAA
PRKAA2	TGATGATGAGCATGTACCTAC	CGACAGAACGATTGAGATATTC
U6	CTCGCTTCGGCAGCACA	AACGCTTCACGAATTTGCGT
miR-124-3p	SSD809230054	SSD809230746
miR-320b	SSD809230296	SSD809230988
miR-320c	SSD809230297	SSD809230989
miR-320d	SSD809230298	SSD809230990

**TABLE 2:** The list of top 20 differentially down-regulated mRNAs arranged from large |log_2_FC| values.

**Gene symbol**	**|log_2_FC|**	***P-*value**	**FDR**	**Chromosome**
KIT	1.434	<1e–07	<1e–07	chr4
CALCRL	1.396	<1e–07	<1e–07	chr2
TNFSF18	1.358	1.00E–07	2.56E–07	chr1
C15orf54	1.322	2.00E–07	4.58E–07	chr15
SULT1B1	1.322	6.00E–06	8.44E–06	chr4
LOC100505865	1.286	3.00E–07	6.34E–07	chr16
SAMD9L	1.286	6.00E–07	1.12E–06	chr7
CFI	1.252	1.00E–07	2.56E–07	chr4
BMX	1.252	1.00E–07	2.56E–07	chrX
MGAT4A	1.252	1.00E–07	2.56E–07	chr2
ZNF521	1.218	1.00E–07	2.56E–07	chr18
C12orf63	1.218	3.00E–07	6.34E–07	chr12
LYVE1	1.218	1.20E–06	2.02E–06	chr11
F2RL2	1.218	2.50E–06	3.86E–06	chr5
C12orf63	1.218	5.20E–06	7.41E–06	chr12
ABCA6	1.184	<1e–07	<1e–07	chr17
TCEAL8	1.184	<1e–07	<1e–07	chrX
GPR116	1.184	<1e–07	<1e–07	chr6
TEK	1.184	<1e–07	<1e–07	chr9
SDPR	1.184	1.00E–07	2.56E–07	chr2

### GO and KEGG Pathways Involved With Differential mRNAs in TC–EC

**FIGURE 2:**
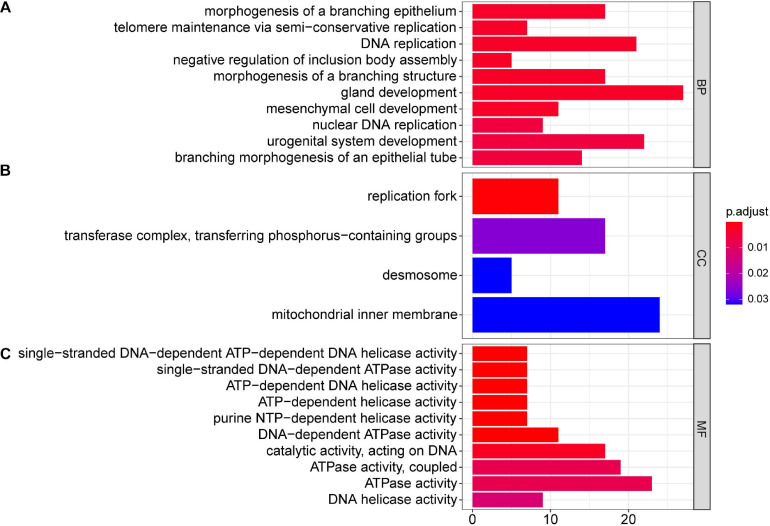
GO analysis of 504 differentially expressed mRNAs in TC–EC interaction, in which gland development, DNA replication, urogenital system development, morphogenesis of a branching epithelium, morphogenesis of a branching structure, branching morphogenesis of an epithelial tube, mesenchymal cell development, nuclear DNA replication, telomere maintenance via semi-conservative replication, and negative regulation of inclusion body assemble are the top 10 biological process **(A)**; mitochondrial inner membrane, transferase complex (transferring phosphorus-containing groups), replication fork, and desmosome are main cell components **(B)**; ATPase activity, catalytic activity acting on DNA, DNA-dependent ATPase activity, and helicase activity are the enriched molecular functions **(C)**. *P* < 0.05 was considered as statistically significant.

Next, we performed GO and KEGG pathway enrichment analyses. The results showed that at the biological process level, the GO functions of differentially expressed mRNAs mainly mediated processes including gland development, DNA replication, and urogenital system development (Figure 2A); at the cell component level, differentially expressed mRNAs were mainly involved in the mitochondrial inner membrane (Figure 2B); and at the molecular function level, differentially expressed mRNAs were mainly enriched in ATPase activity and catalytic activity acting on DNA (Figure 2C).

**FIGURE 3:**
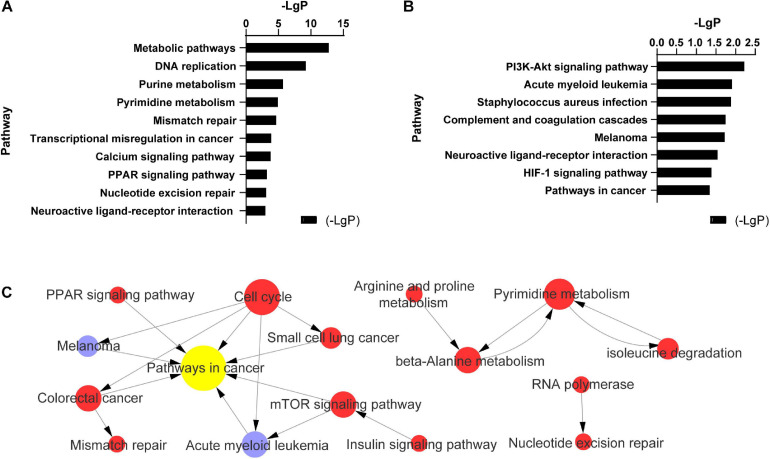
Pathway analysis of 504 differentially expressed mRNAs in TC–EC interaction. **(A)** The most enriched 11 (-LgP > 3) of 40 pathways among up-regulated mRNAs (-LgP > 1). **(B)** The most enriched pathways among down-regulated mRNAs (-LgP > 1). The bar graphs represented the enrichment of these mRNAs. The value of (-LgP) was *P*-value taking the negative logarithm with base 10. **(C)** The interaction between the KEGG pathways of 504 differentially expressed mRNAs. The circle represents the pathway, and the size of the circle represents the degree of the pathway in the network. The larger the degree, the greater the circle, representing more pathway interaction. Lines between pathways represent their interrelationships. Red pathways represent the relative mRNAs that were upregulated, blue pathways represent the relative mRNAs that were downregulated, yellow represents the pathways with both up- and downregulated mRNAs.

In endothelial cells regulated by PC-3M cells, the significantly changed KEGG pathways in which the upregulated mRNAs were enriched mainly included metabolic pathways, DNA replication, purine metabolism, pyrimidine metabolism, and mismatch repair (Figure 3A). The alterative KEGG pathways associated with the downregulated mRNAs in endothelial cells included the PI3K-Akt signaling pathway, acute myeloid leukemia, Staphylococcus aureus infection, Staphylococcus aureus infection, and complement and coagulation cascades (Figure 3B). Integrated analysis showed that the differentially expressed mRNAs mainly regulated pathways in cancer, the cell cycle, pyrimidine metabolism, and the mTOR signaling pathway (Figure 3C).

### Differential mRNA–mRNA Interaction Network

**FIGURE 4:**
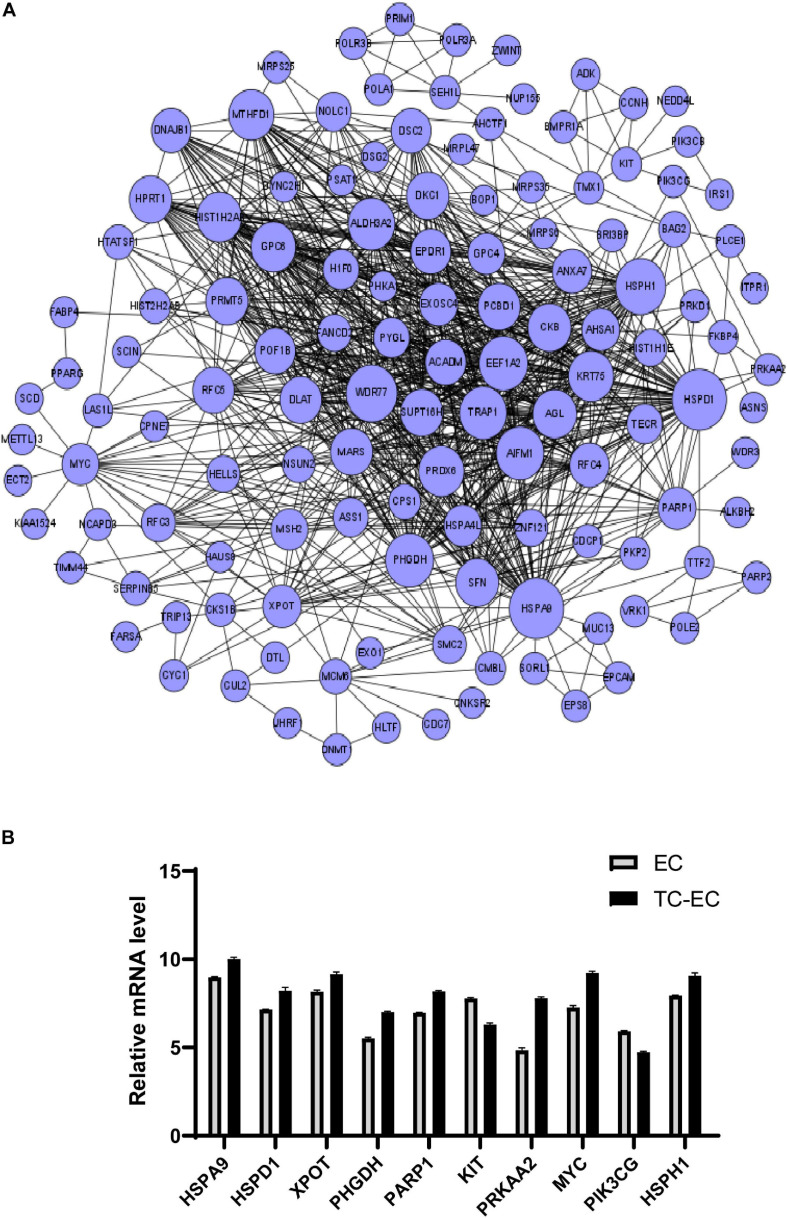
Differential mRNA–mRNA interaction network. **(A)** The network graph of mRNA–mRNA interaction. The circle represents mRNA; the size of the circle represents the betweenness centrality value. The bigger the value, the more signal transmission is involved, indicating that the mRNA has greater ability to regulate mRNA interactions. **(B)** Expression level of the top 10 differential mRNAs between the TC–EC model and EC according to the differential mRNA–mRNA interaction network analysis.

A differential mRNA–mRNA interaction network was constructed to identify the relationship between differentially expressed genes (Figure 4). By computing the betweenness centrality and degree value of each mRNA, we identified the top 10 interacted mRNAs in the mRNA signal transduction network according to their betweenness centrality (Table 3). HSPA9, HSPD1, XPOT, PHGDH, PARP1, KIT, PRKAA2, MYC, PIK3CG, and HSPH1 may have important functions in the gene–gene functional interaction network.

**TABLE 3:** Top 10 betweenness centrality mRNAs in the mRNA–mRNA network.

**mRNA**	**Betweenness centrality**	**Degree**
HSPA9	0.0056	57
HSPD1	0.0034	57
XPOT	0.0031	24
PHGDH	0.0028	44
PARP1	0.0024	23
KIT	0.0023	7
PRKAA2	0.0019	4
MYC	0.0017	21
PIK3CG	0.0017	4
HSPH1	0.0016	48

### mRNA–lncRNA Co-expression Network

**FIGURE 5:**
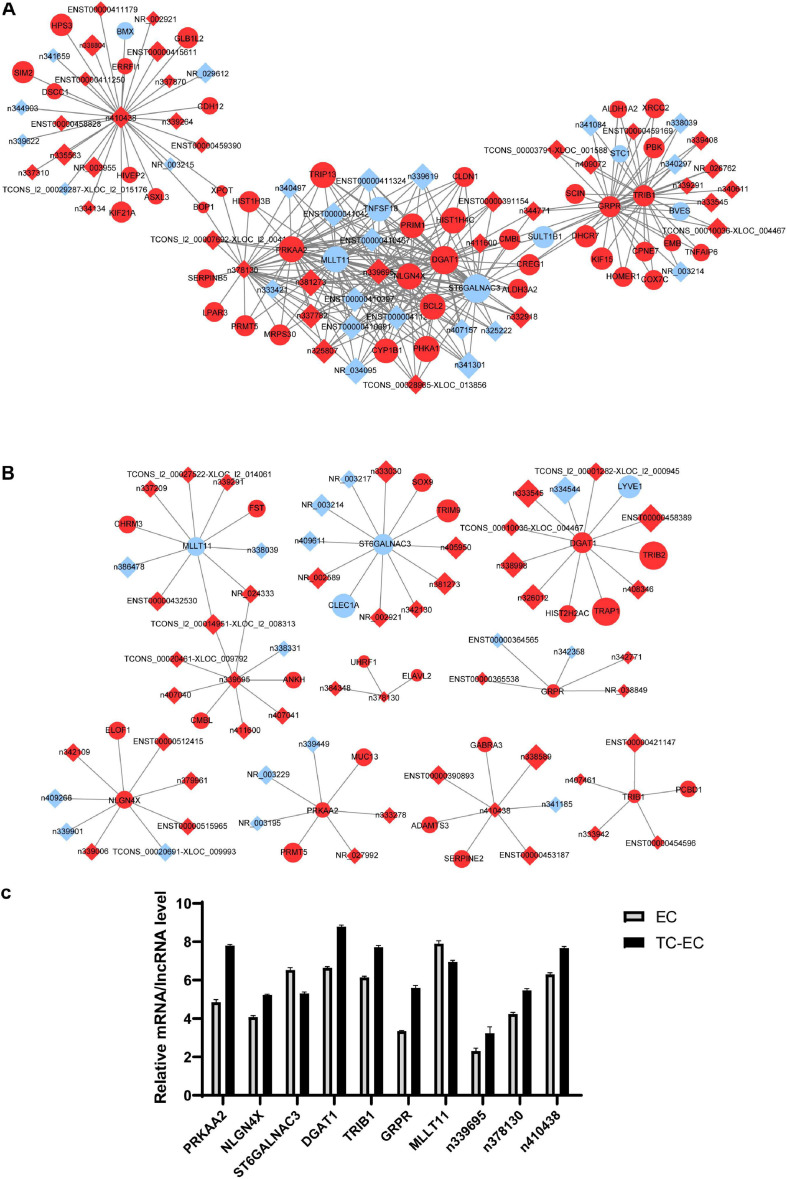
mRNA–lncRNA co-expression network analysis of key mRNAs or lncRNAs in EC alone **(A)** and TC–EC model **(B)**. In the network, the circle represents mRNA, the rhombic represents lncRNA. Red represents the upregulated mRNAs/lncRNAs, blue represents the downregulated mRNAs/lncRNAs. Those mRNA/lncRNAs without interaction relationships were not displayed in the network. **(C)** Expression level of the top 10 differential mRNAs or lncRNAs between the TC–EC model and EC according to the mRNA–lncRNA co-expression network analysis.

Based on the resulting data from the Affymetrix microarray profiling, we constructed an mRNA–lncRNA expression correlation network in both the TC–EC model and EC alone to identify the correlations between mRNAs and lncRNAs. According to the changes of the expression status of differential molecules between the two networks, we identified the top 10 differential mRNAs (PRKAA2, NLGN4X, ST6GALNAC3, DGAT1, TRIB1, GRPR, and MLLT11) and lncRNAs (n339695, n378130, and n410438) in the tumor–endothelium interaction (Table 4). Next, with these 10 genes, we constructed 2 key mRNA–lncRNA interaction networks in the TC–EC model and EC using Cytoscape v3.7.2 (Figure 5).

**TABLE 4:** Top 10 differential mRNAs or lncRNAs between the TC–EC model and EC.

**mRNA/lncRNA**	**Type**	**EC_degree**	**TC-EC_degree**	**|Diffk|**
PRKAA2	Coding	34	7	0.789
NLGN4X	Coding	34	9	0.744
n339695	Non-coding	34	9	0.744
ST6GALNAC3	Coding	36	12	0.733
DGAT1	Coding	36	12	0.733
TRIB1	Coding	30	5	0.722
GRPR	Coding	30	5	0.722
MLLT11	Coding	34	10	0.722
n378130	Non-coding	28	3	0.711
n410438	Non-coding	31	7	0.706

### PRKAA2 mRNA Expression Level Increased and miR-124-3p Expression Decreased in Endothelial Cells Adhered by PC-3M

**FIGURE 6:**
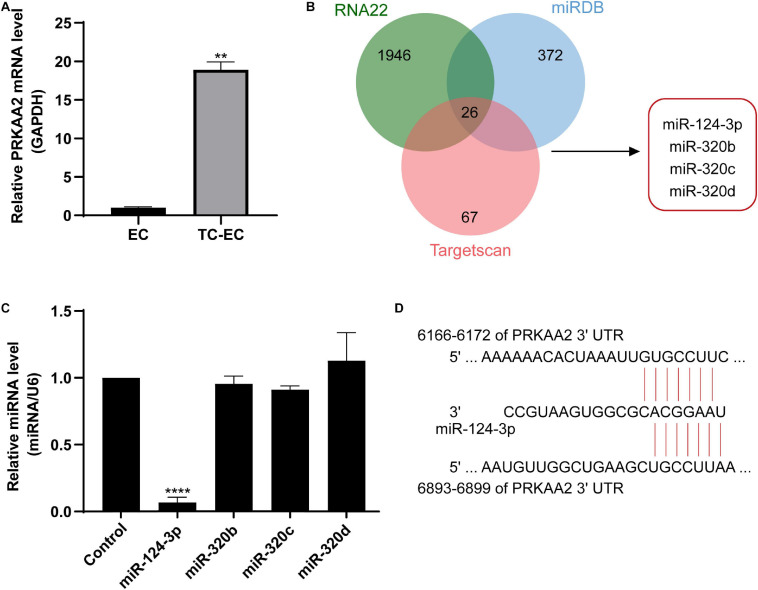
**(A)** The relative expression of PRKAA2 was significantly upregulated in the TC-EC model, compared with EC alone. **(B)** The prediction of miRNAs targeted at PRKAA2, using miRDB, RNA22, and Targetscan database. **(C)** qPCR analysis of relative expression levels of 4 predicted miRNAs targeted at PRKAA2, in which the expression of miR-124-3p was significantly decreased in endothelial cells adhered by PC-3M. **(D)** Predicted binding sites within the 3’UTR region of PRKAA2 with miR-124-3p using the Targetscan database. The data are presented as the means ± *SD*. (*n* = 3, **P < 0.01, ****P < 0.0001, TC-EC vs. EC).

PRKAA2 is a key molecule in endothelial cells adhered by PC-3M. It relates mRNA function with lncRNA according to the mRNA–lncRNA co-expression network. As shown in Figure 6A, the expression of PRKAA2 mRNA in endothelial cells was significantly upregulated after the addition of PC-3M cells, which was consistent with the result of our bioinformatics analysis described above (Figure 6A, *P* < 0.01). In order to explore the upstream mediator of PRKAA2, we used three databases to predict the miRNAs targeted at PRKAA2. As a result, we found 67 miRNAs using the Targetscan database, 372 miRNAs using the miRDB database, and 1715 miRNAs using the RNA22 database, and we obtained 26 miRNAs predicted in all three databases (Figure 6B). We confirmed the expression of 4 common miRNAs (miR-124-3p, miR-320b, miR-320c, and miR-320d) in endothelial cells adhered by PC-3M by performing qPCR. The results showed that miR-124-3p was significantly downregulated in the TC–EC model compared with EC alone (Figure 6C).

Furthermore, we used the Targetscan database to predict the binding sites of miR-124-3p with the 3’UTR region of PRKAA2. The microRNA sequence was 5’-uaaggcacgcggugaaugcc-3’. Targetscan predicted two binding sites of the PRKAA2 3’UTR region, Position 6166–6172 and Position 6893–6899, and the results are shown in Figure 6D.

### Expression Level of PRKAA2 Changed With miR-124-3p Knockdown in Endothelial Cells Adhered by PC-3M

**FIGURE 7:**
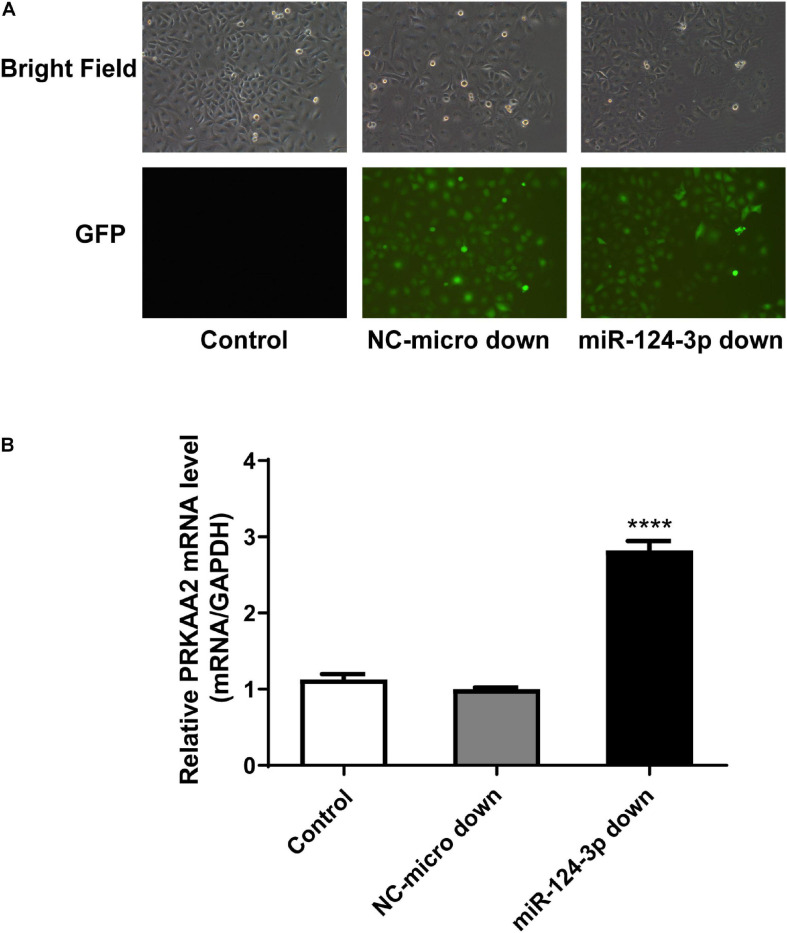
PRKAA2 expression increased with miR-124-3p knockdown in endothelial cells adhered by PC-3M. **(A)** The expression level of miR-124-3p in endothelial cells under fluorescence microscope by green fluorescence protein. BF, bright field; GFP, green-fluorescent protein; NC-miR-124-3p: negative control of lentiviral vector; miR-124-3p-down: miR-124-3p knockdown with lentiviral vector. **(B)** qPCR analysis of relative expression levels of PRKAA2 in HUVECs transfected with LV-miR-124-3p-down. The results show that the expression level of PRKAA2 mRNA in endothelial cells adhered by PC-3M increased greatly with the knockdown of miR-124-3p.

We used lentiviral vector to knock down the miR-124-3p level in endothelial cells adhered by PC-3M cells. The expression level of miR-124-3p was confirmed under a fluorescence microscope by green fluorescence protein (Figure 7A).

Then, using the miR-124-3p knockdown endothelial cell line, we detected the expression of PRKAA2 mRNA. qPCR results showed that the expression level of PRKAA2 mRNA in endothelial cells adhered by PC-3M increased greatly with the knockdown of miR-124-3p (Figure 7B).

### Binding of miR-124-3p With PRKAA2 3’UTR

**FIGURE 8:**
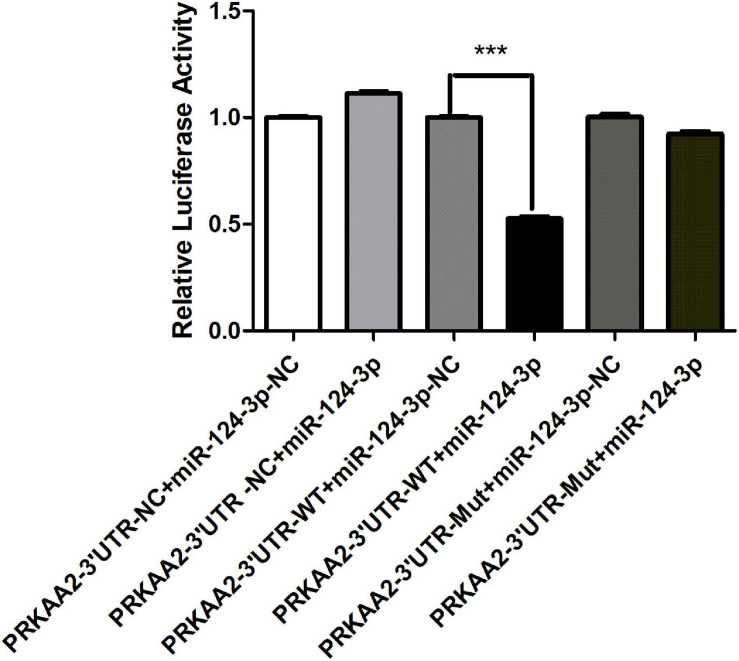
MiR-124-3p targeted PRKAA2 3’UTR in HEK 293T cells. pcDNA3.1(-)-miR124-3p was co-transfected with luciferase reporter constructs containing pGL3-PRKAA2-3’UTR-WT or pGL3-PRKAA2-3’UTR-MUT in 293T cells; Firefly luciferase activity for each construct was normalized to the co-transfected Renilla luciferase construct presented miRNA-124-3p-NC transfected group. The data are presented as the means ± *SD* (*n* = 3, ***P < 0.001, PRKAA2-3’UTR-WT + miR-124-3p vs. PRKAA2-3’UTR-WT + miR-124-3p-NC).

A dual luciferase reporter assay was performed to determine the binding between PRKAA2 3’UTR and miR-124-3p in HEK293T cells. The wild-type 3’-UTR segments or mutated 3’-UTR segments of PRKAA2 and miR-124-3p sequence were cloned into a reporter plasmid downstream from luciferase, and reporter assays were then performed. It was found that after miR-124-3p overexpression, the luciferase activity in the pGL3-PRKAA2 3’UTR-WT-transfected group decreased when compared with the luciferase activity of the pGL3-PRKAA2 3’UTR-MUT group and pGL3-PRKAA2 3’UTR-NC group (Figure 8). The result demonstrated that miR-124-3p could directly bind with PRKAA2 3’UTR.

## Discussion

In this study, we carried out a series of bioinformatics analysis and qPCR validation of differential mRNAs and lncRNAs based on the transcriptome data obtained from Affymetrix GeneChip HTA2.0. The bioinformatics analysis results indicated important biological processes and pathways in tumor–endothelium interaction. By constructing and analyzing a differential mRNA–mRNA interaction network and mRNA–lncRNA expression co-expression network, we identified important mRNAs and lncRNAs in the tumor–endothelium interaction, providing new directions for the study of the mechanisms of tumor–endothelium interaction.

From the GO and KEGG analysis, the biological functions and pathways the differential genes took part in were mainly focused on DNA replication and repair, intracellular substances, and energy metabolism abnormality, especially purine and pyrimidine metabolism abnormality, and signaling pathways related to cell growth and differentiation.

As we know, cancer is a complex multistep process. Metastasis is the leading cause of cancer-related death. When tumor cells enter circulation, their metastatic potential largely depends on having a rapid and efficient way to escape from the blood stream by passing the endothelial barrier (Francesca et al., 2020). Our transcriptome data and analysis showed us that PC-3M tumor cells influenced HUVECs during intravasation and extravasation. Endothelium morphology, DNA replication, mitochondrial components, ATPase activity, and growth-related signaling pathways were changed in endothelial cells adhered by tumor cells.

In the mRNA–lncRNA expression co-expression network, we identified PRKAA2, one of the most important differentially regulated genes in endothelial cells with PC-3M adhesion. PRKAA2 is a catalytic subunit of the AMP-activated protein kinase (AMPK). AMPK is an important energy-sensing enzyme that monitors cellular energy status. Once AMPK is switched on by increases in cellular AMP:ATP ratios, it acts to restore energy homeostasis by switching on catabolic pathways while switching off cell growth and proliferation (Vara-Ciruelos et al., 2019). In cancer cells, studies have shown that AMPK could promote epithelial–mesenchymal transition (EMT) by upregulating Twist 1 and thus promote tumor metastasis (Saxena et al., 2018), and PRKAA2 has been shown to be involved in cell energy metabolism in various cancers. In bladder cancer, loss of PRKAA2 resulted in increased tumor proliferation and larger xenografts (Zhang et al., 2020). AMPK acts downstream of the tumor-suppressor LKB1. The canonical AMP-dependent mechanism of activation has suggested that AMPK might also suppress tumorigenesis. However, once tumorigenesis has occurred, AMPK switches from tumor suppression to tumor promotion (Diana Vara-Ciruelos and Hardie, 2020). We found that PRKAA2 mRNA expression was significantly higher in HUVECs adhered by PC-3M than that in endothelial cells alone. Studies have reported that AMPK can also be switched on by increases in intracellular Ca^2+^, by glucose starvation, and by DNA damage via non-canonical, AMP-independent pathways. In our previous study, we found that the endothelial intracellular Ca^2^ level increased as PC-3M cells were added and contacted to the endothelium, then returned to the original calcium level (Yan Pan et al., 2011). Also, other researchers found that the absence of PRKAA2 could cause aberrant expression and activation of NADPH and consequently cause dysfunction in endothelial cells (Wang et al., 2010). Wang et al. (2010) found that PRKAA2 could protect vascular endothelial cells from oxidative stress by suppressing the NF-κB-mediated expression of NAD(P)H oxidase. Moreover, it has been reported that PRKAA2 could suppress endothelial angiotensin-converting enzyme (ACE) expression via the phosphorylation of p53 and upregulation of miR-143/145 (Kohlstedt et al., 2013).

The metabolism of endothelial cells has recently been recognized as a driving force for tumor angiogenesis. Metabolic pathways, such as glycolysis, fatty acid oxidation, and glutamine metabolism, have distinct, essential roles during vessel formation (Katerina et al., 2018). The finding of the differentially changed gene PRKAA2 in our study is important for energy sensor functions, which could influence endothelial activation when tumor cells contact with endothelial cells. As for its role in tumor cells, AMPK may exert either a positive or negative effect on cancer cell survival depending on the context of cellular stress.

miRNAs are small endogenous non-coding RNA molecules about 22 nucleotides in length that can inhibit the expression of encoded genes and lead to the renewal, conversion, or degradation of mRNA transcription by combining with complementary sequences in the 3’UTR of the target gene through miRNA reaction elements (MREs) (Liu et al., 2017). Therefore, miRNAs also play an important role in cancer as a regulatory factor of key mRNAs. In STK11 mutant lung cancer cell lines (A549 and H838), the miR124-3p-STAT3-PRKAA/AMPKa axis had been reported, which could regulate circHIPK3-induced cell autophagy (Xiuyuan et al., 2020). In our present study, both bioinformatics analysis and qPCR results showed that PRKAA2 is significantly upregulated and its target miRNA miR-124-3p is downregulated in endothelial cells adhered by PC-3M. Luciferase reporter analysis revealed the binding of miR-124-3p with PRKAA2. When the miR-124-3p of endothelial cells were knocked down by shRNA, a significant increase of PRKAA2 level was detected in HUVECs. This indicated that the expression of PRKAA2 is regulated by miR-124-3p. These results also suggest that miR-124-3p and PRKAA2 may play an important role in tumor–endothelial interaction.

Meanwhile, the analysis of lncRNAs in this study is also of great significance. LncRNAs were previously considered to be “transcriptional noise” and thus have no biological function because they do not encode proteins. However, recent studies have shown that lncRNAs are widely involved in the regulation of gene expression and play a significant role in cell proliferation, differentiation, apoptosis, and tumor progression. Based on the analysis of the differential mRNA–lncRNA expression correlation network, we found several lncRNAs that may be closely related to the tumor–endothelium interaction. LncRNA n339695, n378130, and n410438 were upregulated lncRNAs in EC with TC interaction and ranked in the top 10 in the mRNA–lncRNA co-expression network.

According to the ceRNA hypothesis, lncRNAs act as an “miRNA sponge” to moderate miRNA expression and consequently affect mRNA level, forming networks of “lncRNA–miRNA–mRNA” interactions and regulating downstream pathways. Recently, Liu et al. (2019) knocked down lncRNA H19, and they found that cell proliferation and invasion in the ectopic endometrium is inhibited through miR-124-3p targeted suppression of the ITGB3. Therefore, we deduced that it is possible there are several lncRNAs in endothelial cells could regulate the “miR-124-3p/PRKAA2” axis in endothelial cells when it is adhered by tumor cells.

In addition, in the differential mRNA interaction network, HSPA9 and HSPD1 were found to have the greatest ability to regulate mRNA interactions in TC–EC interaction. HSPA9 encodes a member of the heat shock protein 70 gene family, highly expressed in tumor cells and tissues (Wadhwa et al., 2006), and it plays a role in extending cell life, mitochondrial capacity, chaperones, transformation of oncogenes, low differentiation of tumor cells, and stress reactions (Kaul et al., 2007). HSPD1 encodes a member of the heat shock protein 60 gene family, could improve the survival rates of tumor cells through the endogenous apoptotic pathway (Kim et al., 2019), and promotes tumor cell proliferation via the ROS/AMPK/mTOR pathway (Tang et al., 2016). Therefore, HSPA9 and HSPD1 can also be further analyzed as the core genes for the study of tumor–endothelium interaction.

Additionally, another study with metastatic melanoma cells showed that metastatic melanoma cells initiated the disruption of endothelial cell–cell junctions and promoted the formation of intercellular gaps in endothelial monolayers in as few as 10 min of direct coculture. In contrast, non-metastatic melanoma cells do not disrupt the endothelium after direct contact for longer periods of time. A major difference between metastatic and non-metastatic melanoma cells is the ability of the former to interact with the endothelium via the secretion of cytokines and receptor–ligand interactions, which can mediate transendothelial migration (Valastyan and Weinberg, 2011; Aragon-Sanabria et al., 2017; Pantazi et al., 2020). The interaction between endothelial cells with tumor cells is like that of leukocytes. As in the tumor cells, the complex extravasation cascade of leukocytes happens only during inflammatory responses (Muller, 2013). The interaction process happens only during tumor intravasation, extravasation, or angiogenesis and is closely related to the special invasive characteristics of tumor cells (Katia et al., 2020).

## Conclusion

Our study identified aberrantly expressed mRNAs and lncRNAs in endothelial cells, analyzed their biological functions and the signal pathways in tumor–endothelium interaction, and it verified significant upregulation of PRKAA2 mRNA and its targeted miRNA, miR-124-3p, which plays a key role in endothelial cells with tumor adhesion.

## Data Availability Statement

The datasets presented in this study can be found in online repositories. The names of the repository/repositories and accession number is: GSE162957.

## Author Contributions

YP designed the research, completed the experiments, and revised the manuscript. MA analyzed the data and wrote the manuscript. XL and QY guided the experiments. All authors contributed to the article and approved the submitted version.

## Conflict of Interest

The authors declare that the research was conducted in the absence of any commercial or financial relationships that could be construed as a potential conflict of interest.

## Footnotes

1 http://david.abcc.ncifcrf.gov/

2 http://www.kegg.jp/

3 http://mirdb.org/

4 https://cm.jefferson.edu/data-tools-downloads/rna22-full-sets-of-predictions/

5 http://www.targetscan.org/
